# Notes and Notices

**Published:** 1887-07

**Authors:** 


### NOTES AND NOTICES.

Dr. C. E. Chase has removed to 225 Genesee Street, Rochester.

Correction.—June number, page 183, second line from the top, for "results" read *resorts*.

A Bad Habit.—An exchange calls attention to the filthy and injurious habit many have of putting coins in their mouths. It is risky, as serious disease might be contracted.

Dr. Philip Porter, Editor of *The Homœopathic Journal of Obstetrics*, has been appointed Professor of Gynæcology in the Pulte Medical College, of Cincinnati, vice Eaton, resigned.

Dr. Corresta T. Canfield has opened an office in Chicago at 163 State Street, northeast corner Monroe, Rooms, 72 and 73, where she will devote a limited amount of time in receiving those desiring her professional services.

To remove Plaster of Paris bandages, paint a strip across at the desired point of division with a camel's hair pencil dipped into nitric acid or vinegar. The acid will soften the plaster so that it may readily be cut with a knife.

Wanted.—The Dean of one of our "purely homœopathic" institutions wants a cart-load of second-hand hypodermic syringes, for the purpose of drilling his pupils in their use. Weekly lectures are to be given showing there *are* logical reasons for the use of these syringes.

The Registry for Nurses, opened in connection with the Homœopathic Eye and Surgical Institution of Dr. Bushrod W. James, southeast corner Eighteenth and Mount Vernon Streets, Philadelphia, is now fully established. A carefully selected list of trained and other first-class nurses is registered, and their services can be commanded at short notice.

The New Jersey State Homœopathic Medical Society.—The annual meeting was held at Newark on Tuesday, May 3d. The following officers were elected for the ensuing year: Dr. Clarence Willard Butler, of Montclair, President; Dr. J. G. Street, of Bridgeton; Dr. Samuel Long, of New Brunswick, and Dr. E. Rushmore, of Plainfield, Vice-Presidents; Dr. B. H. B. Sleght, of Newark, Recording Secretary; Dr. Wallace McGeorge, of Woodbury, Corresponding Secretary; Dr. F. A. Gile, of Orange, Treasurer; Dr. M. D. Youngman, of Atlantic City, Necrologist; Dr. H. J. Anderson, of Newark; Dr. S. L. Eaton, of Orange; Dr. J. N. Lowe, of Milford; Dr. C. M. Conant, of Orange, and Dr. J. E. Winans, of Lyons Farms, a Board of Census.

THE TRUTH WELL STATED.—Dr. E. M. Harrison gives this confession of faith in the *American Homœopathist.* It is very true and is well stated: "When I was converted to Homœopathy it was an honest conversion. The law to me is infallible. There has been no reason to doubt it, though I made many failures at first with malarial and kindred troubles. I acknowledge the failures to be mine and not those of the law. It was the ignorance of the prescriber, and I allowed no blame to be laid at the door of Homœopathy." Seldom has so much truth been put in so few words; take heed to it, ye who never doubt self, but always decry the law for your every failure.

LECTURES TO NURSES.—Dr. George H. Clark, of Germantown, delivered, May 31st, the seventh of the series of free lectures before the nurses of the Medical, Surgical, and Maternity Hospitals of the Women's Homœopathic Association, of Philadelphia. The lecture was upon Hygiene.

Dr. Franklin Powel, of Chester, Pa., delivered the eighth lecture, June 2d, upon Fever Nursing. What the sick room lacks more than anything else, he said, was a reliable nurse. Such an one was a great relief to the physician, as well as reassuring to the family. Dr. Powel said that the three essentials for a good nurse were: Common sense, tact, and patience.

FROM EMPIRICISM TO HOMŒOPATHY is the suggestive title of *Homœopathic League Tract,* number thirteen. It is well written and tells of the author's gradual progression from empiricism to Homœopathy. It also suggests a point which it may be well to remember: that the progressive allopaths, under the lead of such teachers as Phillips and Ringer, are practicing a sort of mongrel Homœopathy. By the use of these small doses of medicine, given upon the homœopathic principle, these men are much more successful than formerly. So it is that homœopathists of to-day have to contend against a more successful allopath than did Hahnemann and his early followers.

WHO IS THE QUACK?—Dr. Lauder Brunton, whose reputation, such as it is, has been chiefly gained by thousands of experiments on wretched dogs, cats, rabbits, and frogs, which *have not added a single remedy to therapeutics,* yet whose book derives any little value it possesses from his unacknowledged borrowings from the valuable remedies Hahnemann introduced into medicine, now stigmatizes Hahnemann's therapeutics, to which he is so much indebted, as "quackery." It would be more correct to designate his wholesale filchings from Hahnemann as "flat burglary as ever was committed." Has Dr. Brunton so little of a scientific mind that he thinks calling names will do instead of arguments?—*Hom. World.*

CHLORAL ERUPTION.—Dr. Barbillion has described a variety of erythema appearing in patients under the influence of Chloral, upon the administration of Alcohol. The Chloral eruption was first recognized by Jastrowitz, in 1869, and has since been met with by numerous observers. The point to which Dr. Barbillion calls attention is the almost mathematical regularity with which the exanthem appears under certain circumstances. Given a child of from four to eight years of age, who is taking from one-half to one drachm of Chloral daily in repeated small doses, the administration of Alcohol in the form of wine or spirit mixture will cause the appearance of the rash in from fifteen to thirty minutes.—*The Lancet.*

THE JULY NUMBER.—The readers of THE HOMŒOPHATHIC PHYSICIAN are respectfully requested to pardon the delay in the publication of this month's issue. It is unavoidable, owing to the time required to prepare the report of the meeting of the I. H. A., at Long Branch. The verbatim report was very voluminous, and it required much time to transcribe it into long hand and then to condense for our pages.

